# Food Intake of Kansans Over 80 Years of Age Attending Congregate Meal Sites

**DOI:** 10.3390/nu2121297

**Published:** 2010-12-20

**Authors:** Allisha M. Weeden, Valentina M. Remig

**Affiliations:** 1 Department of Health and Nutrition Sciences, Idaho State University, 921 S. 8th Ave STOP 8109, Pocatello, ID 83209, USA; 2 Department of Human Nutrition, Kansas State University, 206 Justin Hall, Manhattan, KS 66506, USA; Email: remig@ksu.edu

**Keywords:** elderly, dietary intake, Modified MyPyramid

## Abstract

As the population of the United States continues to age, it has become increasingly more important to recognize the food intake and eating habits of older adults. The objective of this study was to describe the food group intake, factors predicting food group intake, and the food choices of community-dwelling Kansans, 80 years of age and older who participate in congregate meal programs. Participants completed a short questionnaire querying demographic information, current health status, and dietary supplement use. Participants (*n* = 113) were then followed up via telephone to complete two 24-hour diet recalls. Data were analyzed to determine adequacy of food group intake and mean intake. Regression analyses were used to determine factors predicting intake and frequency analysis established food typically consumed. Female participants were significantly more likely to consume more fruit servings than males. Intake was low for all five of the food groups, especially dairy. Chronic health conditions and dietary supplement use were consistently predictive factors of the amount of each food group consumed.

## 1. Introduction

Adults over 65 years of age, and especially those over age 80, comprise one of the most rapidly growing age groups in the United States. By 2050 the older adult population is predicted to double from the current estimate of 40.2 million to more than 88.5 million [1]. Within the category of older adults is a very diverse population and identifying the nutritional needs and eating habits of population subgroups will become even more important in coming years.

The potential role of nutrition in healthy aging is becoming more evident with each additional research study. It is well documented that the nutritional needs of adults change as they age [2,3]. While caloric needs of older adults decrease with advancing age and reduced physical activity level [2], the need for several nutrients, including calcium and vitamins B6 and D becomes more prominent [3]. To address the changing needs, the Modified MyPyramid for Older Adults has been developed to specifically focus on the nutritional requirements of adults ≥70 years of age [3]. Just like the current USDA MyPyramid, the Modified MyPyramid makes food group recommendations based on caloric and nutrient needs rather than age [3]. Therefore, a single food group recommendation that fits all older adults is not available; the food group recommendations are individualized to meet the needs of each older adult [3].

Previous reports of food group intake have shown older adults tend to consume fewer than the recommended servings for each of the food groups [4,5,6]. Older adults are most likely to meet the recommended intake of fruit [5,7]. Grain group and dairy group recommendations are least likely to be met by older adults [4,5,7].

While several studies have been published on the food intake of older adults, most focus on the younger segment of the older adult population (<75 years of age) and studies investigating the intake of congregate meal participants are even more rare. The objective of this study was to report the food group intake, demographic factors predicting food group intake, and food choices of adults over 80 years of age who regularly attend congregate meal sites.

## 2. Experimental Section

### 2.1. Subjects

Between June 2007 and January 2008, community-based older adults were recruited to participate in a cross-sectional study investigating dietary supplement use and dietary intake. Participants were recruited from randomly selected senior centers throughout Kansas. Of the originally recruited 374 participants, 113 were identified as being eligible for the current analysis. Inclusion criteria required participants to be at least 80 years of age who completed both 24-hour diet recalls. The cutoff age of 80 years is consistent with the age categories used in the presentation of data from the National Health and Nutrition Examination Survey (NHANES) [4]. Details of sampling and recruitment were presented elsewhere [8]. All participants provided informed written consent on the day of enrollment and study procedures were approved by the Institutional Review Board at Kansas State University.

### 2.2. Data Collection and Analysis Procedures

At the time of enrollment, all participants completed a questionnaire developed to collect demographic information, current health status, and dietary supplement use. Participants were then contacted via telephone on two separate occasions to complete a 24-hour diet recall. All 24-hour diet recalls were conducted by a trained registered dietitian using the multi-pass technique [9].

Food group intake and serving size were categorized using the dietary analysis software Nutritionist Pro (Version 3.1, 2006, Stafford, Texas) and the USDA’s MyPyramid.gov website [10]. Data from the two 24-hour diet recalls was averaged and caloric needs for each participant were estimated using age, height and weight [11]. Mean food group intake from the 24–hour diet recalls was compared to the recommended food group intake level corresponding to the estimated caloric needs of each participant. The recommended number of servings for each food group varied greatly among participants. Therefore, a recommended intake level has not been included with the results. Data has been reported for each of the five food groups: grains, vegetables, fruit, dairy, and meat/beans. Protein foods including meat, fish, poultry, eggs, and beans are all included in the group labeled meat/beans.

### 2.3. Statistical Methods

All statistical analyses were performed using SPSS (Version 15.0, 2006, SPSS, Inc., Chicago, IL). Frequency, mean, and range analyses were used to determine demographic distribution and food group intake. Chi-square was used to determine differences in food group intake between males and females. Multiple linear regression was used to assess significance of predictors on the amount of each food group consumed. Backward step method was used to eliminate non-significant variables and create better models. Variables included in the original model were age, gender, rural/urban location, marital status, education level, alcohol use, activity level, diagnosis of a diet related chronic disease (including type 2 diabetes, high cholesterol, hypertension, and heart disease), and dietary supplement use. All categorical variables were coded as indicator variables: gender (0 = female, 1 = male), rural/urban location (0 = community < 50,000; 1 = community > 50,000), marital status (0 = divorced/widowed, 1 = married), alcohol use (0 = no alcohol consumption, 1 = alcohol consumption), diet related chronic disease (0 = no, 1 = yes), and dietary supplement use (0 = no, 1 = yes). Activity (coded 1–4, 1 = not active, 4 = 5 or more days/week) and education (coded 1–5, 1 = less than high school, 5 = postgraduate degree) were coded as ordinal values. Actual age values were used in the models. Multi-collinearity of each model was checked using variance inflation factors. Normality was assessed using the Kolmogorov-Smirnov test and residual plots. Frequencies were calculated using the number of cups or ounces of a specific food to determine foods most often consumed by study participants. A *p-*value of <0.05 was considered statistically significant.

## 3. Results and Discussion

### 3.1. Study Participants

Study participants were primarily widowed, white females (*n* = 83) living in rural communities (<50,000 inhabitants). Participant age ranged from 80–96 years. Mean age was 84.8 years (84.3 years for males and 85.0 years for females). Despite most participants reporting self-perceived health status as excellent/very good to good, 93.8% of participants reported having been diagnosed with at least one chronic disease. The most commonly reported chronic diseases were arthritis (58.4%), high blood pressure (54.9%), and diabetes (30%). Participants frequently reported the use of dietary supplements. A general multi-vitamin, multi-mineral (58.4%) and calcium supplement (56.3%) were the most frequently used dietary supplements; herbal supplement use was reported by only 6.2% of participants. Approximately half of the participants reported that they participated in activities like walking or senior aerobics classes at least three times per week. Further characterization of participants is listed in Table 1.

**Table 1:** Demographics and characteristics of participants.

Characteristic		% (n)
Gender		
	Male	26.5 (30)
	Female	73.5 (83)
Race/Ethnicity		
	White, non-Hispanic	96.5 (109)
	Black/Hispanic/Native American	3.5 (4)
Marital Status		
	Married	21.2 (24)
	Widowed/Divorced/Single	78.8 (89)
Yearly Income		
	<$6,000	47.8 (54)
	$6,000–$24,000	19.5 (22)
	Did not disclose	32.7 (37)
Education (*N* = 111)		
	<High school	15.0 (17)
	High school	36.3 (41)
	Some college/vocational school	39.8 (45)
	College graduate	7.1 (8)
Current Health Status		
	Excellent/very good	30.1 (34)
	Good	43.4 (49)
	Fair/poor	26.5 (30)
Alcohol Use (*N* = 108)		
	Never consume	86.7 (98)
	Consumes weekly	8.8 (10)
Tobacco Use (*N* = 110)		
	Never used	62.8 (71)
	Current/former user	34.5 (39)
Dietary Supplement Use		
	Yes	85.8 (97)
Activity (*N* = 111)		
	Not active	23.9 (27)
	1–2 days/week	23.9 (27)
	3–4 days/week	27.4 (31)
	>5 days/week	23.0 (26)
Community Size		
	≤50,000	64.6 (73)
	≥50,001	35.4 (40)

### 3.2. Food Group Intake

The percent of participants achieving adequate intakes for each of the five food groups is presented in Table 2 and the mean intake from each food group based on estimated caloric value are presented in Table 3. Overall, the number of participants achieving adequate intake for each of the food groups was low; no participant in the current study met the recommendations for all five food groups. Food intakes from the dairy group were most likely to be low. Only 3.5% of participants (1 female and 3 males) met the recommended three cups per day from the dairy group. Participants were most likely to achieve adequate intakes from the fruit group and meat/bean group. The only statistical difference between gender and adequate food group consumption was with fruit intake. Females were statistically more likely to meet the daily fruit requirement than males (*p* = 0.039).

**Table 2:** Percent of participants achieving adequate intakes of each of the five food groups.

	% Within Males	% Within Females	% of Total Sample
**Grain**	16.7	18.1	17.7
**Vegetable**	10.0	24.1	20.4
**Fruit**	16.7	39.8 *	33.6
**Dairy**	10.0	1.2	3.5
**Meat & Beans**	16.7	24.8	29.2

* *p* < 0.05.

As expected, the food group intake for the older adults surveyed was lower than recommended levels [4,5,6,7,12]. When MyPyramid was released in 2005, one of the changes made was to redefine serving size. Focus group research found the term serving used with the Food Guide Pyramid to be confusing to consumers [13]. To minimize confusion, the recommended servings of each food group were replaced with a recommended intake expressed in ounces and cups [13]. Due to the change it is difficult to make numerical comparisons between the results of the current study to those of previously published research that compared adequate food group intake to the servings recommended in the Food Guide Pyramid.

In general, previous studies also indicated that adults greater than 80 years of age report intake of grains below recommended levels [5,7]. Intake of dairy foods also tends to be low among older adults. In a study of rural North Carolina adults, Vitolins *et al.* reported a mean dairy intake among 80+ year olds to be approximately 50% of the intake recommended by the Food Guide Pyramid. This intake is slightly higher than in the current study, where most participants only consumed one-third of the recommended daily intake. Nutrition knowledge was not measured in the current study, but with the development of the new MyPyramid in 2005, the food group recommendations changed [3] and older adults may not be aware of the increased need for dairy products and calcium. Another possibility is that older adults have a taste aversion to milk or prefer not to drink milk. In fact, only 22% of the fluid milk consumed by participants was consumed with congregate meals.

**Table 3:** Mean food consumption of each food group based on estimated caloric value.

	Estimated caloric needs					
	1400 (rec. intake) ^a^	1600 (rec. intake)	1800 (rec. intake)	2000 (rec. intake)	2200 (rec. intake)	2600 (rec. intake)
	Mean intake ± S.D.					
**Grain (oz)**	4.06 ± 1.35 (5)	3.61 ± 1.57 (5)	3.51 ± 1.54 (6)	4.88 ± 1.55 (6)	4.04 ± 1.63 (7)	4.60 ± 1.30 (9)
**Vegetable (cups)**	1.72 ± 0.70 (1.5)	1.35 ± 0.67 (2)	1.12 ± 0.70 (2.5)	1.21 ± 0.68 (2.5)	1.55 ± 1.15 (3)	2.00 ± 0.86 (3.5)
**Fruit (cups)**	1.73 ± 1.04 (1.5)	1.26 ± 0.79 (1.5)	1.03 ± 0.79 (1.5)	1.04 ± 0.58 (2)	1.64 ± 1.14 (2)	0.85 ± 0.41 (2)
**Dairy (cups)**	0.88 ± 0.43 (3)	1.03 ± 0.64 (3)	0.67 ± 0.55 (3)	1.33 ± 0.68 (3)	2.06 ± 0.99 (3)	1.75 ± 1.05 (3)
**Meat/Beans (oz)**	3.94 ± 1.60 (4)	3.88 ± 1.76 (5)	3.93 ± 2.19 (5)	4.31 ± 1.87 (5.5)	4.84 ± 1.33 (6)	3.50 ± 0.80 (6)

^a^ Recommended intakes as suggested by the USDA MyPyramid.

Nearly 30% of the participants in the current study met the recommended intake of meats and beans; slightly lower than 36% of participants meeting the recommended intake in the Rural Nutrition and Oral Health Study [6]. The low fruit and vegetable intake reported in the current study is consistent with previous reports [6,14,15]. During the 24-hour diet recalls, many older adults surveyed indicated that they believed the meal received at the congregate meal site was a “good, well-balanced meal” and because of the quality of the congregate meal, nutrition was not as important at home.

Additional trends and beliefs were also observed during the 24-hour diet recall interviews and subsequent data analysis. First, many participants reported decreased feelings of hunger. Second, participants frequently indicated that activity levels had decreased and therefore resulted in the belief that less food was needed. Third, most of the older adults participating in the current study followed the same general meal pattern. Nearly all participants ate breakfast, though it varied in size, followed by a “large” lunch at the congregate meal site. This observation is supported by the findings of the *Pilot Study: First National Survey of Older Americans Act Title III Service Recipients*, which estimated that congregate meals provide half to two-thirds of daily calories consumed for nearly 50% of congregate meal participants [16]. The evening meal was usually much lighter and often consisted of half a sandwich, small bowl of soup, or leftovers from the noon meal. The taking of leftovers from the congregate meal site does create potential food safety issues as food may not be refrigerated or reheated properly. Fruit was often consumed with the evening meal, but vegetables were less likely to be included with the evening meal. The trends observed from the 24-hour diet recall interviews had not previously been reported and were not supported by statistical analysis. Nonetheless, it is important to note the general trends observed, especially when establishing why older adults often report food group intakes below recommended levels. The results presented here may be specific to Kansas, but previous analysis has shown participants in the current study were characteristically similar to other congregate meal attendees throughout the United States [8,17].

### 3.3. Factors Predicting Food Group Intake

Demographic and health factors predicting food group intake are reported in Table 4. Following the order of that table, increased activity level was the only variable significantly predictive of grain intake (*p* = 0.018). For each day participants participated in physical activity, 0.334 more ounces of grains were consumed. Use of tobacco was associated with lower intake of the grain group (*p* = 0.090). Participants who reported the use of tobacco consumed 0.866 ounces less of grains than participants who do not use tobacco. Previous research had indicated individuals who are more active are more and did not use tobacco have a healthier lifestyle that includes improved dietary intake [18].

**Table 4:** Demographic and health factors predicting food group intake.

Food Group	Factors	B	β	*P*-value
**Grain**				
	Activity	0.334	0.235	0.018
	Tobacco use	−0.866	−0.168	0.090
**Vegetable**				
	Age	−0.038	−0.192	0.057
	Diet related chronic disease	−0.353	0.164	0.033
**Fruit**				
	Education level	0.164	0.177	0.079
	Diet related chronic disease	−0.326	−0.173	0.085
**Dairy**				
	Gender	0.410	0.262	0.006
	Rural/urban	−0.257	−0.180	0.059
**Meat/Beans**				
	No statistically significant model			

Higher vegetable intake was predicted by younger age (*p* = 0.057) and no diet related chronic disease (*p* = 0.033). For each year in age, participants consumed 0.038 cups less of vegetables. Participants reporting a diet related chronic disease consumed 0.353 fewer cups of vegetables than participants without a diet related chronic disease. There is strong evidence that individuals who consume greater quantities of fruits and vegetables have lower blood pressure [19]. Higher vegetable intake had previously been associated with reduced incidence of chronic disease [20].

Factors included in the model predicting higher fruit intake were education (*p* = 0.079) and diet related chronic condition (*p* = 0.085). For each increase in education level, participants consumed 0.164 cups more of fruit than those with lower education levels. Participants with a diet related chronic condition consumed 0.332 cups less fruit than those without a diet related chronic condition. In general, higher consumption of more healthful foods had been associated with higher education and lower incidence of chronic disease [21,22].

Higher dairy intakes were predicted by male gender (*p* = 0.006), and rural/urban (*p* = 0.059). Males consumed 0.41 cups more of dairy products than female participants. Male gender had been previously associated with greater dairy intake [4]. Participants residing in rural areas consumed 0.257 cups more of dairy products than their urban counterparts. The increased dairy consumption among rural participants may be a reflection of the agrarian society found in rural Kansas.

None of the currently selected variables produced a statistically significant model in the prediction of meat/bean group intake.

### 3.4. Food Choices of Older Adults

Over 34% of the grain ounces consumed by participants in the current study were yeast breads/dinner rolls. The second most commonly consumed grain product was ready-to-eat breakfast cereals (10%). Rounding out the top five grain products were cookies (8.6%), desserts like cake or pie (8.3%), and hot cereals (7.9%). Oatmeal was the most commonly consumed hot cereal. Previous analysis of the 2001-2002 NHANES (analysis included all NHANES participants) found that the most commonly consumed grain products were ready-to-eat cereals, hot cereals, and yeast breads [23]. The high intake of cookies and desserts was consistent with the data presented by Vitolins *et al.*, which indicated that adults 85+ years of age consumed much greater quantities of sweets than their younger counterparts [5].

As expected, white potatoes were the most commonly consumed vegetable (24.4%), followed by mixed vegetable dishes (10.8%) and tomatoes (10.2%). Peas and carrots were a mixed vegetable dish commonly served by the congregate meal sites. The most commonly consumed vegetable in the United States is the white potato [13,23,24]. Tomatoes and vegetable medleys have also been previously reported as top sources of vegetables in the United States [23].

The most common fruit choices were bananas (15.3%), orange juice (12.7%), and peaches (10.3%). Other fruit juices (8.9%) and apples (8.6%) were also frequently reported. The primary fruit selections of the current population were relatively soft fruits and may have been influenced by the age of the population investigated. Apples, pears, and bananas have been previously reported as significant contributors to fruit intake based on 2001–2002 NHANES data [23]. The same NHANES analysis also found fruit juice, especially orange juice and grapefruit juice, to be a major contributor to fruit intake [23].

Fluid milk made up over 60% of dairy product intake in the current study; reduced fat or 2% fat milk was the most commonly selected variety. Other frequently consumed dairy products included cheese (16.4%) and ice cream (10.6%). In the 2001–2002 NHANES study, most milk servings were in the form of fluid milk and reduced fat was most commonly reported [23]. Ice cream was consumed more frequently in the current study than in previous reports [23] and may reflect the time of year that data was collected.

Chicken (18.5%), hamburger/ground beef (11.8%), and other beef including roast beef, steak, *etc.* (11.6%) were the most commonly consumed meat products. The most commonly consumed meats reported in the 2001–2002 NHANES study included hamburgers, sausage, hot dogs, bacon, and ribs [23]. While the participants in the current study did consume sausage, hot dogs, and bacon, the reported intake was infrequent. The high chicken intake can be attributed to congregate meal sites frequently serving chicken. Beef was also popular at congregate meal sites, and as fast food hamburgers. The beef intake in the current study was higher than previously reported and may be due to the location of the study and availability of beef in Kansas.

### 3.5. Strengths and Weaknesses

Strengths of the study include the population studied. A unique element of the current study is that all participants reported eating meals at congregate meal sites. The study also included a relatively large number of adults greater than 80 years of age and incorporated individuals living in both rural and urban settings. Also, the use of 24-hour diet recalls allowed for observations of dietary patterns.

Weaknesses of the study were a lack of minority participants. The original study did have minorities, but most were younger were thus excluded from the current analyses. Also, all subjects were volunteers and therefore some individuals who were not willing to complete the questionnaire or telephone interviews may have been excluded. Dietary data collected was dependent upon the ability of individuals to accurately recall foods consumed from the previous day. Finally, the cross-sectional nature of the study and use of 24-hour diet recalls prevent further extrapolation of the data to include the length of time the observed dietary practices have been in place.

## 4. Conclusions

The overall food group intake of participants in the current study was low. Comments made by participants indicating they believed only one well-balanced meal was needed each day may indicate a need for nutritional education. This study supports the position held by the American Dietetic Association to encourage older adults to participate in food service programs like the congregate meal sites and for dietitians to be more involved with the development of nutritional education programs designed for older adults [25]. Further study is needed to determine the nutritional knowledge of the 80+ year old population within the United States. Additional analysis of the reliance on congregate meal sites for the primary source of nutrition regardless of income should be conducted. By determining the use and reliance on the meal sites, we can better determine the best way to address the nutritional needs of adults as they progress through old age.
